# Supplementary material for: A tipping point in refreezing accelerates mass loss of Greenland's glaciers and ice caps
Source: Nat Commun. 2017 Mar 31;8:14730. doi: 10.1038/ncomms14730 (PMC5380968; doi:10.1038/ncomms14730)
Supplement: Supplementary Information — Supplementary Figures, Supplementary Tables, Supplementary Discussion and Supplementary References [file ncomms14730-s1.pdf]

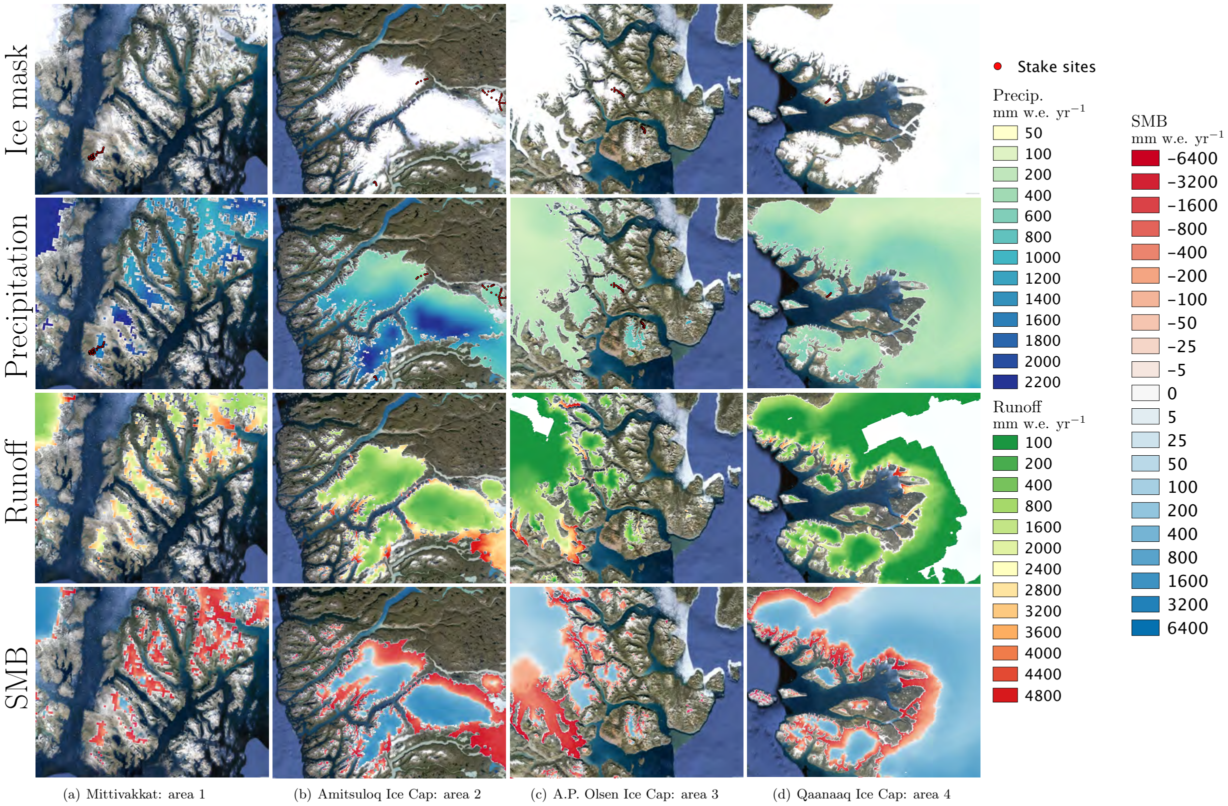

Supplementary Figure 1: **High-resolution GICs SMB patterns.** Example maps of ice mask, downscaled total precipitation (solid and liquid), runoff and reconstructed SMB v1.0 in a) Mittivakkat region (black box 1 in Fig.1), b) Amitsuloq and Sukkertoppen ice caps (black box 2), c) Zackenberg region (black box 3) and d) Qaanaq ice caps (black box 4). Red dots locate the selected stake transects shown in Fig. 1.

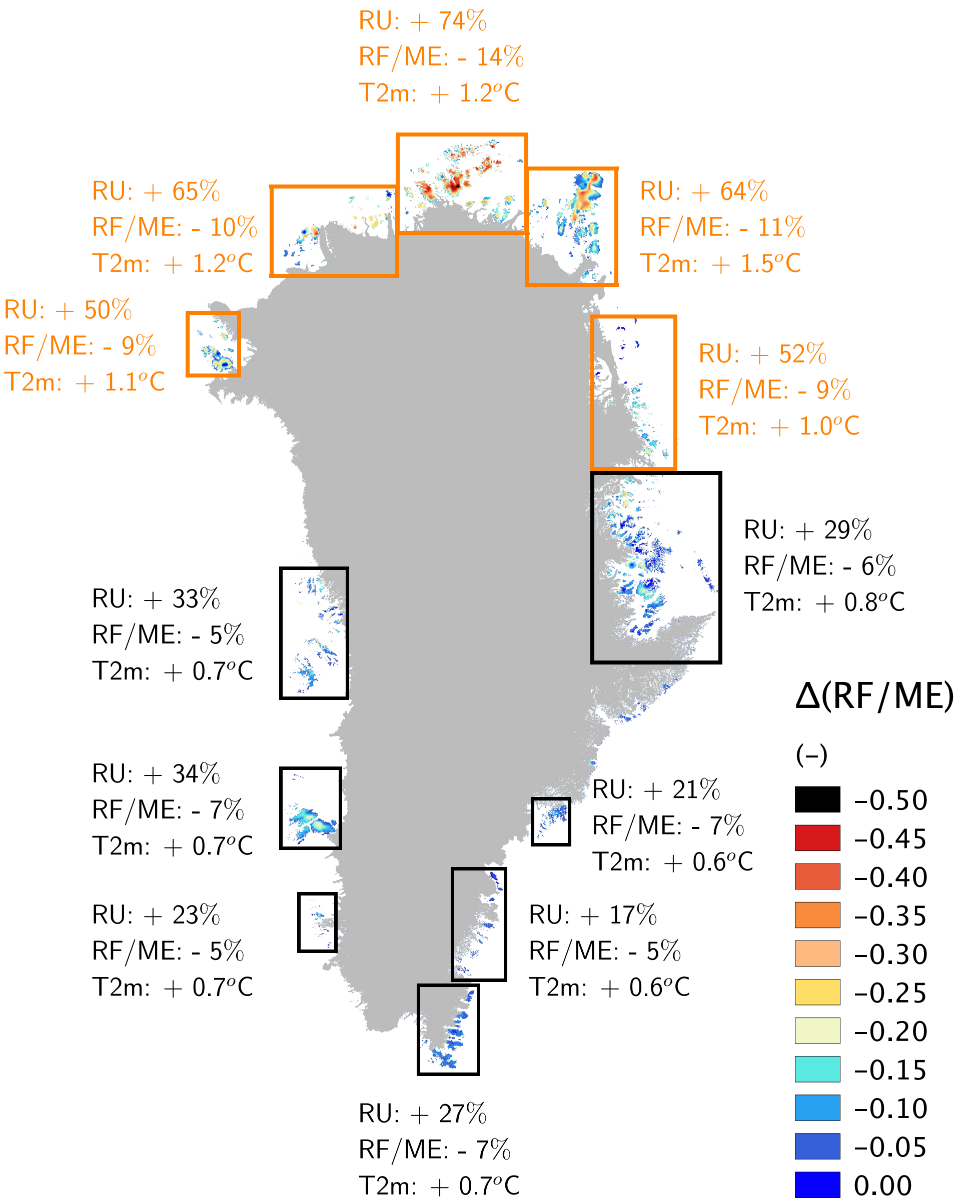

Supplementary Figure 2: **Drivers of the latitudinal contrast in runoff intensification.** Regional differences in annual mean refreezing-to-melt ratio between 1997-2015 and 1958-1996. The change in runoff, refreezing-to-melt ratio and near-surface temperature is estimated for twelve different regions in north (orange boxes) and south Greenland (black boxes). The contiguous GrIS is displayed in grey.

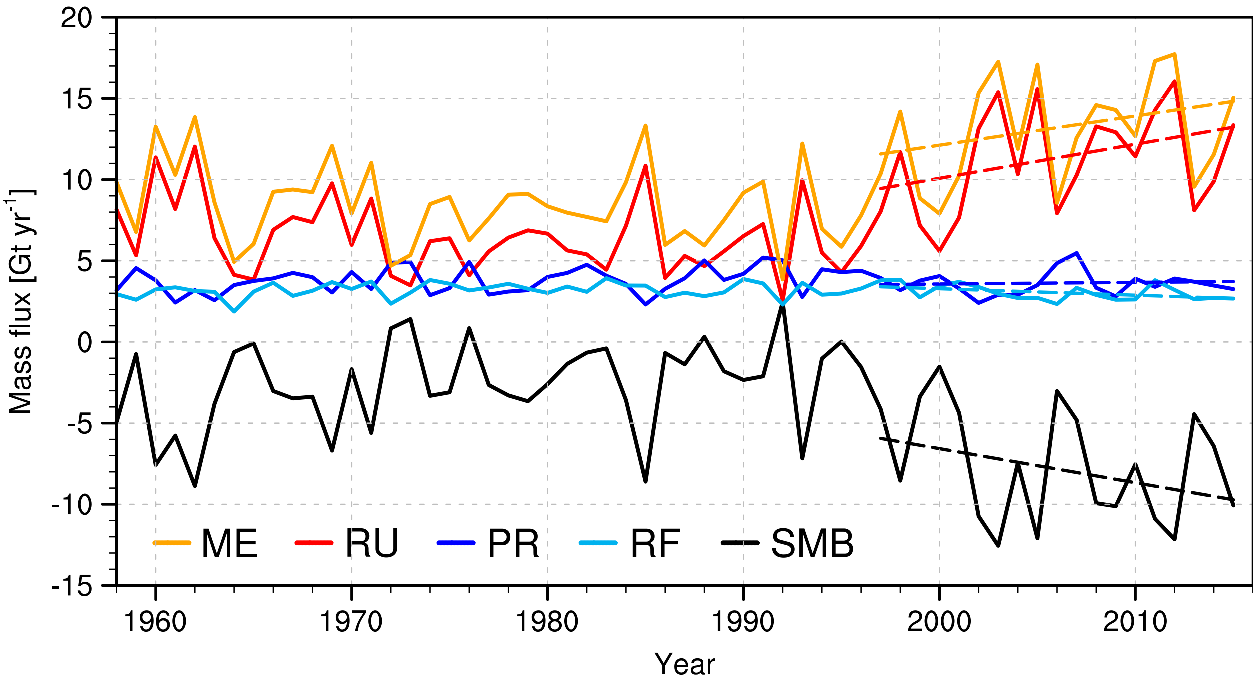

Supplementary Figure 3: **Mass flux evolution in the Hans Tausen region.** Time series of annual cumulative SMB components over the Hans Tausen ice cap and surroundings (Black box 5 in Fig. 1) for the period 1958-2015. Trends in SMB components are represented as coloured dashed lines (1997-2015).

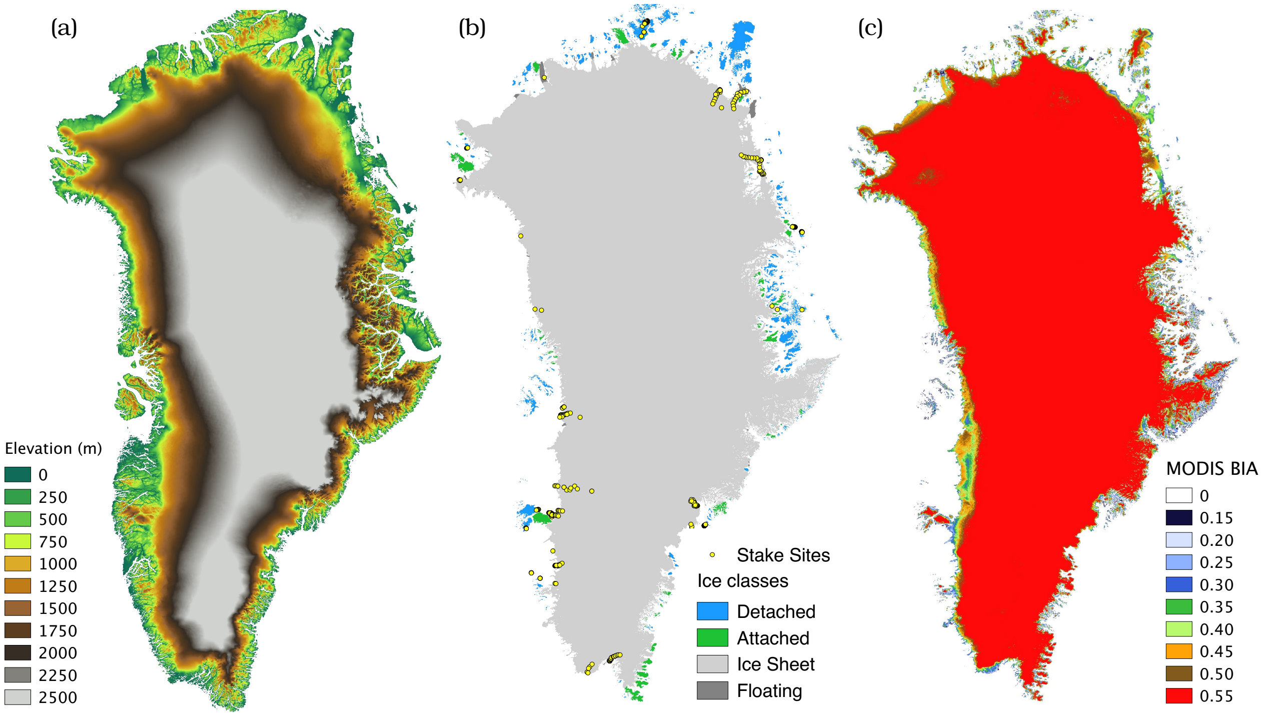

Supplementary Figure 4: **High-resolution Greenland surface properties.** a) Elevation (m) derived from the GIMP DEM at 1 km; b) PROMICE ice mask showing the contiguous ice sheet (light grey), GICs attached to the ice sheet but not dynamically connected (green), physically detached GICs (blue) and floating glacier tongues (dark grey); c) MODIS bare ice albedo at 1 km (2000-2015). Stakes sites used for SMB evaluation are displayed in yellow in b).

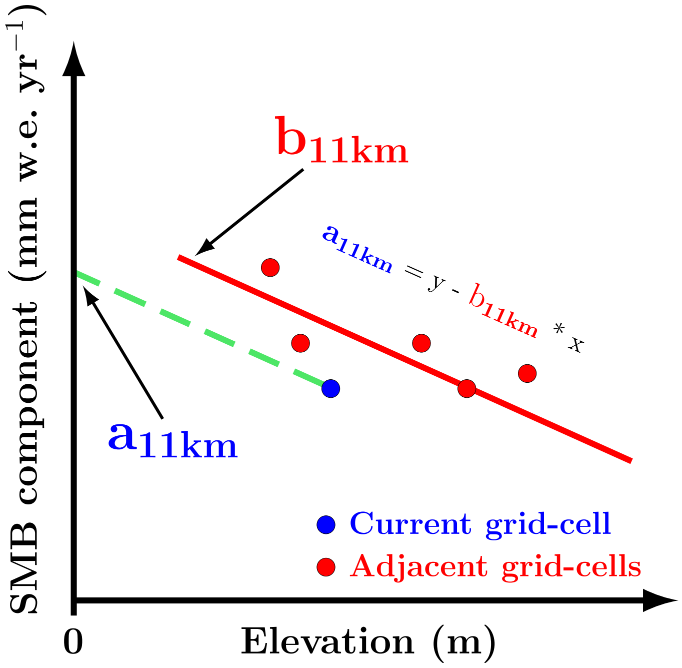

Supplementary Figure 5: **Elevation-dependent downscaling procedure.** b_{11km} and a_{11km} are respectively the daily local estimates of the SMB components regression to elevation and the SMB components value at mean sea level obtained on the RACMO2.3 grid at 11 km. The red line corresponds to the regression (b_{11km}) calculated using the current grid cell (blue dot) and the adjacent ones (red dots). The dashed green line applies the regression slope to the current grid cell to estimate a_{11km} (from Noël et al., 2016).

Mean	Units	SMB	RU	PR	ME	RF
1958-1996	Gt yr^{-1}	-11.3 $\pm$ 15.7	67.5	57.5	95.1	36.0
1997-2015	Gt yr^{-1}	-36.2 $\pm$ 15.7	92.8	57.9	119.5	36.0
1958-1996	mm w.e. yr^{-1}	-139 $\pm$ 193	829	706	1168	442
1997-2015	mm w.e. yr^{-1}	-445 $\pm$ 193	1140	711	1468	442

Trend	Units	SMB	RU	PR	ME	RF
1958-1996	Gt yr^{-2}	-0.01 $\pm$ 0.22	0.06 $\pm$ 0.17	0.06 $\pm$ 0.09	0.26 $\pm$ 0.16	0.20 $\pm$ 0.04
1997-2015	Gt yr^{-2}	-1.12 $\pm$ 0.63	1.09 $\pm$ 0.64	-0.02 $\pm$ 0.13	0.66 $\pm$ 0.61	-0.47 $\pm$ 0.08

Supplementary Table 1: **GICs SMB average and trends.** Greenland's GICs-integrated annual mean cumulative SMB components (top) and trends (bottom) for the periods 1958-1996 and 1997-2015. SMB components include runoff (RU), total precipitation (PR), total melt (ME) and refreezing (RF).

Mean	Units	SMB	RU	PR	ME	RF
1958-1996	Gt yr^{-1}	378.6 ± 52.5	324.9	746.3	556.4	238.3
1997-2015	Gt yr^{-1}	255.0 ± 52.5	453.6	752.1	728.9	285.5
1958-1996	mm w.e. yr^{-1}	193 ± 27	165	380	283	121
1997-2015	mm w.e. yr^{-1}	130 ± 27	231	383	371	145

Trend	Units	SMB	RU	PR	ME	RF
1958-1996	Gt yr^{-2}	1.11 ± 1.62	0.19 ± 0.90	1.36 ± 1.08	1.26 ± 1.12	1.09 ± 0.39
1997-2015	Gt yr^{-2}	-10.41 ± 4.02	6.86 ± 3.69	-3.49 ± 2.57	8.77 ± 5.40	1.83 ± 2.41

Supplementary Table 2: **GrIS SMB average and trends.** GrIS-integrated annual mean cumulative SMB components (top) and trends (bottom) for the periods 1958-1996 and 1997-2015. SMB components include runoff (RU), total precipitation (PR), total melt (ME) and refreezing (RF).

Gt yr^{-1}	1971-09[1]	1993-09[1]	2003-08[2]	2003-09[2]	2005-09[3]
Recent studies	-21 ± 10	-37 ± 10	-41 ± 17	-38 ± 7	-56 ± 10
Current study	-17 ± 16	-30 ± 16	-40 ± 16	-40 ± 16	-39 ± 16

Supplementary Table 3: **GICs mass loss estimates.** Annual mean mass loss estimated for different periods and derived from previous studies (top, *1* Marzeion et al., 2012; 2* Bolch et al., 2013; 3* Gardner et al. 2013*) and the downscaled dataset v1.0 (bottom). Uncertainties in the downscaled dataset are obtained by integrating the SMB mean bias (see Methods) over the GICs area.

Supplementary Discussion

We select five GICs regions (black boxes in Fig. 1) to highlight the downscaled SMB dataset and demonstrate its ability to resolve realistic patterns over small glaciers. Supplementary Fig. 1 shows Landsat satellite imagery for four of these regions, with superimposed downscaled 1958-2015 average total precipitation (solid and liquid), runoff and SMB (v1.0).

Mittivakkat (region 1; 65.69^{o}N, 37.77^{o}W; Fig. 1 and Supplementary Fig. 1a) is a small glacier in southeast Greenland. This region experiences relatively large precipitation ($\sim$1600 mm w.e. yr^{-1}) combined with pronounced summer runoff ($\sim$2000 mm w.e. yr^{-1}). The downscaled SMB shows realistic gradients (Fig. 1). Fig. 1 and Supplementary Fig. 1b show two ice caps in southwest Greenland, Sukkertoppen and Amitsuloq (region 2; 66.20^{o}N, 52.10^{o}W). These ice caps have well-defined accumulation zones owing to pronounced precipitation maxima in their interior and runoff being restricted to the margins. Interestingly, the area with large ablation in the southeastern corner of the downscaled product mirrors the region of dark bare ice exposed at the surface in the satellite image.

Supplementary Figs. 1c and d show the Zackenberg (74.64^{o}N, 21.45^{o}W) and Qaanaaq regions (77.50^{o}N, 69.17^{o}W) in northeast and northwest Greenland, respectively (regions 3 and 4 in Fig. 1). Compared to southern Greenland, these regions are characterized by a relatively cold and dry climate resulting in less marginal runoff, narrowing the ablation zone. The modelled SMB profile for the southern slope of Qaanaaq ice cap shows a systematic positive bias of $\sim$0.5 m w.e. (Fig. 1), potentially as a result of inaccurate representation of the narrow ablation zone.

Supplementary References

1. Marzeion, B. A., Jarosch, A. H. & Hofer, M. Past and future sea-level change from the surface mass balance of glaciers. *The Cryosphere* **6**, 1295 – 1322 (2012).

2. Bolch, T. *et al.* Mass loss of Greenland's glaciers and ice caps 2003-2008 revealed from ICESat laser altimetry data. *Geophysical Research Letters* **40**, 875 – 881 (2013).

3. Gardner, A. S. *et al.* A reconciled estimate of glacier contributions to sea level rise: 2003 to 2009. *Science* **340**, 852 – 857 (2013).
